# Potential Risk Factors for Cutaneous Squamous Cell Carcinoma include Oral Contraceptives: Results of a Nested Case-Control Study

**DOI:** 10.3390/ijerph7020427

**Published:** 2010-02-03

**Authors:** Maryam M. Asgari, Jimmy T. Efird, E. Margaret Warton, Gary D. Friedman

**Affiliations:** 1 Division of Research, Kaiser Permanente Northern California, Oakland, CA 94612, USA; E-Mails: maryam.m.asgari@nsmtp.kp.org (M.M.A.); margaret.m.warton@nsmtp.kp.org (E.M.W.); gary.friedman@nsmtp.kp.org (G.D.F.); 2 Department of Dermatology, University of California San Francisco, San Francisco, CA 94115, USA; 3 Center for Health of Vulnerable Populations, Office of the Dean, School of Nursing, University of North Carolina, Greensboro, NC 27402, USA; 4 Department of Health Research and Policy, Stanford University School of Medicine, Stanford, CA 94305, USA

**Keywords:** squamous cell carcinoma, oral contraceptives, risk factors, epidemiology

## Abstract

Recently, a population-based case-control study observed a 60% increased odds ratio (OR) for cutaneous squamous cell carcinoma (SCC) among women who had ever used oral contraceptives (OCs) compared with non users (95% confidence interval (CI) = 1.0–2.5). To further characterize the putative association between OC use and SCC risk, we conducted a nested case-control study using a large retrospective cohort of 111,521 Kaiser Permanente Northern California members. Multivariable conditional logistic regression was used to estimate ORs and CIs adjusting for known and hypothesized SCC risk factors. Pre-diagnostic OC use was associated with a statistically significant increased OR for SCC in univariate analysis (OR = 2.4, CI = 1.2–4.8), with borderline statistical significance in multivariable analysis (CI = 2.0, CI = 0.91–4.5). Given the high incidence of SCC in the general population and the prevalent use of OCs among women in the United States, there is a need for more large, carefully designed epidemiologic studies to determine whether the observed association between OC use and SCC can be replicated and to better understand the etiologic basis of an association if one exists.

## 1. Introduction

Cutaneous squamous cell carcinoma (SCC) is the second most common malignancy in the United States [1] and its incidence is steadily rising [2,3]. Although surgical excision is often curative, cutaneous SCCs can metastasize and become fatal, especially in immunocompromised patients [4,5]. Treatment can cause significant disfigurement and morbidity and accounts for high health-care expenditure [6]. Known risk factors associated with SCC can be divided into host-related and environmental factors. Host-related factors include innate pigmentary characteristics [7,8], sun sensitivity [9], precursor lesions [10], genetic predisposition (xeroderma pigmentosa, epidermoplasia verruciformis) [11], and immunologic factors [12]. Environmental factors include exposure to physical agents, the best characterized of which is ultraviolet light [13–15]. Both UVA and UVB light initiate and promote carcinogenesis [16] and immunosuppression [17]. Chronic cumulative exposure to ultraviolet radiation has been established as a risk factor for SCC [7,9,18]. Other physical agents include ionizing radiation [18], chemical agents (polycyclic aromatic hydrocarbons, arsenic, and nitrosamines) [11], medications used for immunosuppression, chronic inflammation (ulcers, sinus tracts), trauma (burns and scars), and viruses (certain types of human papillomavirus) [20,21] also have been implicated.

Epidemiological studies suggest that individuals with cutaneous SCC are more likely to develop other malignancies compared with individuals who have no history of non-melanoma skin cancer (NMSC) [22–24]. Additionally, in patients who have a history of NMSC, there is an increased incidence of and mortality from leukemia, non-Hodgkin’s lymphoma, and cancers of the lung, bladder, breast, testis, salivary gland, small intestine, and pharynx [25]. According to several recent epidemiological studies, a history of NMSC may be associated with 20 to 30 percent increased mortality from another type of cancer [23,25]. Understanding exposures that predispose individuals to cutaneous SCCs also may shed light on risk factors for these other types of malignancies. Recently, a population-based case-control study reported a statistically significant increased risk estimate for SCC among oral contraceptive (OC) users than non users [26]. Using an established cohort of Kaiser Permanente Northern California (KPNC) members with data on self-reported pre-diagnostic characteristics, we performed a nested case-control study to further examine the putative association between OC use and SCC in the context of other environmental and host-related risk factors.

## 2. Methods

### 2.1. Study Population

The source population consisted of members of KPNC who had completed at least one Multiphasic Health Checkup (MHC) between July 1964 and August 1973. The MHC, initiated in 1964, was a voluntary, comprehensive health evaluation that included a detailed self-administered MHC questionnaire (MHCQ), a standardized physical examination, and a group of specialty examinations [27]. The MHC has been used for numerous risk factor studies [28–30].

This study focused on the program’s medical center in Oakland, which provided health care to many of the area’s adult subscribers [31] and had computer-stored surgical pathology records starting in 1974. These pathology records were coded by histology using Systematized Nomenclature of Human and Veterinary Medicine (SNOMED) codes [32]. Tumor registry records, mostly complete from 1969, allowed us to identify patients with prior histologically confirmed cancers (other than NMSCs) within at least five years of their initial SCC diagnosis. These patients (~19%) were not included in the selection of patients with SCC in order to minimize the immediate effects of surveillance bias, treatment effects, disease-induced immunosuppression, and the possibility of residual cancer. However, patients with more distant self-reported physician diagnosed cancers, ascertained from their earliest MHCQ, were included in the selection of SCC cases and analyzed as a covariate in our statistical model.

We examined the histology codes of the 111,521 MHC cohort members enrolled in Oakland between 1964 and 1973 and identified individuals who had a histology code for a cutaneous SCC during follow-up from 1974 through 1995. For each case, we randomly selected up to five controls who were members at the time of case diagnosis and who were matched for age at the time of examination (±2 years), gender, residential postal zip code, and year of health checkup (±5 years). Excluded from the analysis (cases and controls) were 157 (4.5%) non-Caucasians, 12 (0.34%) participants diagnosed with genital SCCs and 82 (2.4%) participants with missing values on the MHCQ for smoking status, occupational exposures, history of cancer, birth control pill use (if female), eye color, or aspirin use. A total of 392 (76%) SCC cases had two or more matched controls. The final analysis dataset consisted of 516 cases and 1,690 controls. This study was approved by the Institutional Review Board of KPNC.

### 2.2. Risk Factors

Information on possible SCC risk factors was obtained from each subject’s earliest MHCQ and from information recorded by the MHC staff (eye color). If data were missing from the earliest MHCQ, they were obtained from later pre-diagnostic MHCQs, if available. Maximum follow-up was 22 years, with a mean of 19.2 years. Host-related factors were defined as those that are inherent to participants. Environmental factors were defined as those derived from external exposures such as OC use, smoking, drug exposure and sun-exposure proxies, including leisure time activities and occupational exposure. These risk factors are shown in Table 1. To assess education levels, participants were asked to check the highest grade completed in school, and these responses were grouped into those who had completed education up to high school, and those who had completed some college or trade school. As a marker for pigmentation, eye color was divided into four categories: brown, blue/gray, green, and other. Participants also were asked about the number of hours they spent each day in the past year in leisure time activities, as well as exercise.

The MHCQ inquired about occupational exposure by asking, “In the past year, have you worked in a place where you were often or daily around:” followed by a list of 11 categories: (1) chemicals, cleaning fluids, or solvents, (2) insect or plant sprays, (3) ammonia, chlorine, ozone, or nitrous gases, (4) engine exhaust fumes, (5) plastic or resin fumes, (6) lead fumes or metal fumes, (7) silica, sandblasting, grinding, or rock drilling, (8) X-ray or radioactivity, (9) extreme heat, (10) asbestos, cement, or grain dusts, or (11) ultraviolet radiation (UV). All 11 categories of occupational exposures were examined for possible association with SCC risk.

Finally, we ascertained self-reported regular use of three classes of medications postulated *a priori* to be associated with SCC: (1) aspirin (“six tablets or more of aspirin including Empirin, Anacin, or Bufferin almost every day”) hypothesized to decrease SCC risk [33,34]; (2) “cortisone type medication,” hypothesized to increase SCC risk due to immunosuppression [35] and (3) oral contraceptives (birth control pills), hypothesized to increase SCC risk due to its association with anogenital SCCs [36].

### 2.3. Statistical Analysis

Odds ratios (ORs) were used as the measure of association for binary outcome variables and were computed using conditional logistic regression [37]. Normal theory approximation using Wald’s method was used to determine the 95% confidence intervals (CI) for the OR estimates (ORs, hereafter referred to as risk) [38]. All variables were included in the multivariable model except for occupational exposures not associated with SCC risk in the univariate model. Analyses for oral contraceptive use were limited to women. Statistical analyses were performed using SAS, version 9.1 (SAS Institute Inc., Cary, NC).

## 3. Results

A total of 516 participants with SCC included 321 men and 195 women. The mean age was 71.4 years (standard deviation 11.3, range 33–97). The majority (62%) of SCCs were diagnosed on the head and neck consistent with previous reports [3,39]. The remaining tumors were located on the upper extremities (11%), trunk (9%), lower extremities (9%), and other non-genital skin (10%).

### 3.1. Host Factors Associated with SCC

Marital Status. Risk for SCC was increased among women who were currently or ever married, but CIs spanned unity.

Education. Women who completed education beyond high school were at increased risk for SCC than those less educated. Although positive in direction, the effect was diminished among men.

Body Mass Index. Risk for SCC was not increased for any category of body mass index.

Eye Color. Blue/gray eye color was associated with an increased risk for SCC. Participants that were classified as having “other” eye color also had a higher risk for SCC. We assumed that the “other” category was more likely to represent light color eyes, such as hazel. Also, among women, but not men, green eye color was associated with an increased risk for SCC.

Personal History of Cancer. Men, but not women, who self-reported a history of cancer had an increased risk of SCC.

### 3.2. Environmental Factors Associated with SCC

Cigarettes. There was no significant association of SCC with cigarette smoking history among men, whether comparing never smokers to former or current smokers. A borderline increased risk for SCC was observed for women who were former but not current smokers.

UV Exposure. Although the MHCQ did not specifically inquire about sun exposure habits, it obtained information on occupational UV exposure and time spent in leisure activities or exercise, which we reasoned would be the majority of exposure to UV. Men who spent two or more hours a day in leisure activities had a higher risk for SCC than those who spent less than two hours, and the risk estimate increased with increasing time spent in leisure activities. An increased risk for SCC was observed among women who spent 5–7 hours a day in leisure activities but the effect was not statistically significant for other levels of exposure. Except for a borderline significant effect for women who exercised 2–4 hours a day, this variable was not associated with an increased risk for SCC when controlling for other variables in the model. Among men, occupational exposure to UV radiation was the strongest predictor of SCC risk in our multivariable model.

Oral Contraceptive Use. We found a statistically significant risk for SCC in regular users of OC in the univariate model (Table 1). In the multivariable model, the OR changed little, although the confidence interval widened yielding an association that was no longer statistically significant.

Aspirin and Cortisone. Women who consumed >six aspirin tablets a day had a decreased risk for SCC but CIs were wide. Cortisone type medications were not associated with SCC risk.

Other Occupational Exposures. Exposure to dust (asbestos, cement or grains) was associated with decreased SCC risk among men. Only two women in our study reported this exposure and neither were diagnosed with SCC. The remaining nine occupational exposure categories were not associated with future SCC.

## 4. Discussion

We observed a borderline statistically significant association between oral contraceptive use and subsequent SCC risk among women in our cohort. If not merely due to chance or study bias, a possible explanation is that oral contraceptives, which contain estrogen (ethinylestradiol) and/or progesterone (progestin or synthetic progesterone-like compounds), alter the serum estradiol/progesterone ratio, which may influence the oncogenic potential of cutaneous squamous cells [40,41]. Estrogen receptors are present in normal skin [42]. However, SCCs are not believed to express significant amounts of sex hormones, suggesting that an association between OC use and SCC may be mediated through non-sex hormone pathways [43]. One possible pathway is *p53*, as estrogen appears to inhibit the actions of this tumor suppressor [44]. Inactivation of the *p53* gene is believed to play a pivotal gatekeeper role in SCC carcinogenesis [45–47]. Risk also may be indirectly increased through interactions with polymorphisms in nucleotide excision repair (NER) genes including *Xeroderma pigmentosum group D* (*XPD*) [26]. Another possibility is that women who use birth control pills may have certain lifestyle factors, such as increased sexual activity, which may make them more likely to harbor HPV. Infection with some HPV types has been implicated in the pathogenesis of cutaneous SCCs in immunocompetent hosts [1,21,48–52]. The association between birth control use, HPV risk and cervical SCC has been reported [36,53–55]. A similar association could hold for cutaneous SCC.

To the best of our knowledge, only one published paper to date has studied the association between OC use and SCC [26]. Overall, OC users had a 1.6 adjusted odds ratio (OR) for SCC (CI = 1.0–2.5). ORs also were higher among women who last used OCs ≥ 25 years before diagnosis (OR = 2.1, CI = 1.2–3.7), and within group ORs increased with duration of use (OR for ≤ 2 years, 1.7; CI = 0.9–3.5; OR for 3–6 years, 2.6; CI = 1.0–6.5; OR for ≥7 years, 2.7; CI = 0.9–8.5, *P*_trend_ = 0.01). Our results support these previously published findings.

The epidemiology of SCCs has been difficult to characterize because conventional national registries, such as the Surveillance, Epidemiology, and End Results (SEER) [56] program exclude NMSCs. The unique advantages of the Kaiser Permanente Northern California (KPNC) setting are that it closely simulates the surrounding population serving nearly one-third of the insured population of Northern California and it houses an electronic database that captures information on all pathology specimens received for examination, allowing for thorough and accurate capture of incident SCCs. Recall bias was not a concern in our study since OC use was recorded prior to the diagnosis of SCC.

One potential limitation of this study is residual confounding due to indirect measurement of sun exposure, a known risk factor for SCCs [7,9,18]. We used occupational UV and time spent in leisure activities, or exercise, as surrogate markers for sun exposure reasoning that sun exposure comes from two primary sources: time spent in the sun for leisure/exercise and time spent in the sun related to one’s occupation. Among men and to lesser degree women, the strength of association with leisure time activities increased with time, supporting the assumption that time spent in leisure activities is correlated with UV exposure. However, leisure time activity may have been an inexact surrogate measure of sun exposure as the prompting examples (“Hobby, TV, *etc.*”) given for the question on the MHCQ were vague and may have been interpreted as activities that did not involve sun exposure. Nor did the question differentiate between sun and non sun related leisure time activities. Similarly, the MHCQ did not specify the type or duration of exercise, or whether the exercise was performed indoors *versus* outdoors. Surprisingly, time spent in exercise was slightly protective among men. Although this may seem counterintuitive, it is conceivable that exercise conveys health benefits that may offset the negative effect of UV exposure. Furthermore, some participants may have underreported occupational sun exposure since they might not have known that “ultraviolet radiation” was a surrogate term for “sun exposure.” While our surrogate measures of sun exposure were inexact, there is no inherent reason to believe that sun exposure is associated with OC use and thus unlikely to have affected the risk estimates for this variable.

Attenuation of risk estimates may have occurred if some control subjects had SCC diagnosed outside of the KPNC system. This is unlikely because KPNC is a comprehensive healthcare system and members would have to pay out-of-pocket for services received outside the health plan. Also, the exposures that we studied were obtained at a single point in time and were not measured over the entire study follow-up period.

The possibility remains that women who take oral contraceptive medicines may be more likely to interact with the healthcare system and increased risk for SCC may be due to detection bias. However, most SCC is diagnosed years after discontinuing OC use. Women with a history of OC use may have differentially received greater screening for cervical cancer. In our analysis, we adjusted for level of education, a factor believed to influence screening behavior.

In the current study, OC use was crudely measured as “ever/never” exposed and did not include information on dose, duration of OC use, pill composition, or serum estradiol and progesterone levels. The potency and overall dose of OCs have changed over time. Our results reflect the use of earlier compositions of OCs when hormone doses were considerably higher and may no longer hold for present day OCs. Nonetheless, this study is an important addition to the literature as the use of OCs was recorded prior to skin cancer diagnosis.

Although KPNC is generally reflective of the broader population in Northern California [57,58], there are some differences that may have introduced uncontrolled factors regarding ethnicity or behavior. In addition, individuals who self-selected for the MHCQ may have differed from the larger KPNC population on these as well as other distinguishing characteristics that were not directly measured and could have introduced selection and/or detection bias. However, these factors probably did not affect the overall validity of our case *versus* control comparisons.

Occupational exposure to dust from asbestos, cement, or grain was included as a potential confounding factor in our model based on our hypothesis that occupational dust may coat the skin to form a physical barrier to UV light. Previous reports of occupational risk and keratinocyte tumors have not noted this specific association [59]. Exposure to agricultural dusts have been associated with decreased lung cancer risk [60] suggesting a possible anticancer effect independent of an interaction with UV light. However, a healthy worker survival effect and reduced smoking among farmers may have been a more plausible explanation for the reduced risk observed among men in our study [61]. A limitation of our analysis was that the MHCQ grouped all three types of dust into one category and the association of each with SCC might differ.

The MHCQ also did not differentiate between types of cancer in the self-reported personal history of cancer question. Thus, it was not possible to determine if prior history of self-reported cancer and SCC risk was due to a personal history of NMSC *versus* other cancers. NMSC is the most common cancer in the United States [4] and history of NMSC is a major risk factor for a new primary SCCs [62]. In our study, risk was higher only among men who self-reported a history of cancer and may reflect a higher incidence of NMSC among this group [63,64]. Although our main effects multivariable analysis was adjusted for self-reported personal history of cancer, it is possible that the resulting estimate for OC use was affected by residual confounding.

We did not detect a consistent association between smoking and SCC risk as has been reported by some studies [65–68]. Our finding may reflect a different study population which may be susceptible to different gene-environment interactions. Or it may be due to our simple smoking history classification (*i.e.*, never, former, current) which did not account for pack years smoked, filtered *versus* unfiltered, and other detailed smoking information. However, the possibility remains that smoking does not increase SCC risk as was observed in a large occupational cohort study [69]. Given the uncertainty of our smoking variable, we cannot rule out residual confounding in our observed association between OC use and SCC.

The association of SCC with innate pigmentary factors, such as light eye color, is well established [7,8] and is supported by our data. Our results indicate that environmental exposures which were used as surrogate markers for UV exposure (occupational UV in the past year and time spent in leisure activities) also were highly correlated with SCC risk, as expected. Similarly, our finding of an association between higher education and SCC risk has been previously reported [19,70,71]. Education level may affect SCC risk through socioeconomic status leading to differences in lifestyle and health-seeking behavior. Those individuals with more education may have higher socioeconomic status, allowing them to take mid-winter vacations in sunny locations, leading to higher SCC risk due to more frequent episodic sunburns. Alternatively, higher education also may lead to detection bias if more educated individuals are more likely to seek health care. Further studies on the mechanisms underlying the association between education and SCC risk are needed.

In summary, we observed a borderline statistically significant increased SCC risk with use of oral contraceptives similar to that reported in a recent case-control study. On the present evidence, it is not possible to definitively answer the question of how OC use influences SCC risk, if such an association exists, or to favor any specific hypothesis. If confirmed in future studies, these results will lead to new insight in the etiology of SCCs.

## Figures and Tables

**Table 1.** Conditional logistic regression models comparing cases and controls.*

**Characteristic**	**SCC n (%)**	**Control n (%)**	**Univariate OR (95% CI)**	**Multivariate٤ OR (95% CI)**
**Host Factors**				

Marital status۶				
Never married				
Male	20 (6)	33 (3)	1.0 Referent	1.0 Referent
Female	7 (4)	26 (4)	1.0 Referent	1.0 Referent
Married				
Male	237 (74)	791 (78)	0.62 (0.31–1.3)	0.71 (0.33–1.5)
Female	118 (61)	402 (59)	1.1 (0.45–2.8)	1.5 (0.54–4.3)
Separated/divorced/widowed				
Male	16 (5)	65 (6)	0.66 (0.27–1.7)	0.77 (0.29–2.1)
Female	43 (22)	142 (21)	1.3 (0.50–3.5)	1.8 (0.61–5.5)

Education (years of college)۶				
High school or less				
Male	130 (41)	459 (45)	1.0 Referent	1.0 Referent
Female	94 (48)	368 (54)	1.0 Referent	1.0 Referent
Any college				
Male	176 (55)	(47)	1.4 (1.02–1.9)	1.3 (0.95–1.8)
Female	92 (47)	262 (39)	1.8 (1.2–2.6)	1.9 (1.2–2.8)

Body Mass Index (kg/m^2^)۶				
<20 (underweight)				
Male	5 (2)	17 (2)	0.64 (0.20–2.0)	0.58 (0.16–2.1)
Female	25 (13)	60 (9)	0.95 (0.55–1.6)	1.0 (0.55–1.9)
20–25 (healthy)				
Male	157 (49)	348 (34)	1.0 Referent	1.0 Referent
Female	109 (56)	303 (45)	1.0 Referent	1.0 Referent
≥25 (overweight)				
Male	150 (47)	434 (43)	0.78 (0.60–1.02)	0.78 (0.59–1.04)
Female	58 (30)	169 (25)	0.94 (0.63–1.4)	1.1 (0.69–1.6)

Eye color (right)				
Brown				
Male	72 (22)	307 (30)	1.0 Referent	1.0 Referent
Female	39 (20)	218 (32)	1.0 Referent	1.0 Referent
Green				
Male	6 (2)	38 (4)	0.73 (0.29–1.9)	0.63 (0.23–1.8)
Female	17 (9)	36 (5)	2.7 (1.3–5.5)	2.6 (1.2–5.5)
Blue/gray				
Male	184 (57)	495 (49)	1.6 (1.1–2.0)	1.6 (1.1–2.2)
Female	99 (51)	322 (47)	1.7 (1.1–2.6)	1.9 (1.2–2.9)
Other				
Male	59 (18)	171 (17)	1.6 (1.1–2.5)	1.5 (0.98–2.4)
Female	40 (21)	103 (15)	2.0 (1.2–3.5)	2.2 (1.2–3.9)

Personal history of cancer~^□^				
No				
Male	290 (90)	975 (96)	1.0 Referent	1.0 Referent
Female	167 (86)	583 (86)	1.0 Referent	1.0 Referent
Yes				
Male	31 (10)	36 (4)	2.8 (1.6–4.9)	2.8 (1.5–5.0)
Female	28 (14)	96 (14)	1.1 (0.69–1.8)	0.93 (0.55–1.6)

**Environmental Factors**				

Smoking status (cigarettes)				
Never				
Male	123 (38)	366 (36)	1.0 Referent	1.0 Referent
Female	105 (54)	397 (58)	1.0 Referent	1.0 Referent
Former				
Male	105 (33)	384 (38)	0.90 (0.65–1.2)	0.91 (0.64–1.3)
Female	36 (18)	104 (15)	1.6 (0.99–2.6)	1.6 (0.97–2.8)
Current				
Male	93 (29)	261 (26)	1.0 (0.74–1.4)	1.0 (0.71–1.5)
Female	54 (28)	178 (26)	1.0 (0.69.1.6)	1.0 (0.64–1.6)

Ultraviolet radiation§				
No				
Male	312 (97)	1005 (99)	1.0 Referent	1.0 Referent
Female	193 (99)	675 (99)	1.0 Referent	1.0 Referent
Yes				
Male	9 (3)	6 (1)	4.4 (1.5–13)	5.0 (1.7–15)
Female	2 (1)	4 (1)	3.0 (0.51–18)	2.5 (0.34–20)

Leisure time activities (# hours/day)†۶				
0–1				
Male	32 (10)	112 (11)	1.0 Referent	1.0 Referent
Female	19 (10)	89 (13)	1.0 Referent	1.0 Referent
2–4				
Male	207 (64)	651 (64)	1.1 (0.71–1.8)	1.3 (0.76–2.1)
Female	121 (62)	425 (63)	1.4 (0.82–2.6)	1.4 (0.76–2.7)
5–7				
Male	37 (12)	76 (8)	1.6 (0.84–2.9)	1.8 (0.92–3.6)
Female	23 (12)	46 (7)	2.7 (1.2–5.8)	2.7 (1.1–6.4)
>7				
Male	10 (3)	15 (1)	3.3 (1.1–9.5)	3.8 (1.2–12)
Female	2 (1)	7 (1)	1.4 (0.24–7.7)	1.1 (0.18–6.7)

Exercise(# hours/day)⋄۶				
0–1				
Male	176 (55)	504 (50)	1.0 Referent	1.0 Referent
Female	100 (51)	384 (57)	1.0 Referent	1.0 Referent
2–4				
Male	93 (29)	283 (28)	0.89 (0.65–1.2)	0.91 (0.65–1.3)
Female	57 (29)	150 (22)	1.6 (1.1–2.4)	1.5 (0.95–2.3)
5–7				
Male	10 (3)	41 (4)	0.66 (0.30–1.4)	0.67 (0.29–1.5)
Female	7 (4)	22 (3)	1.5 (0.58–3.8	1.3 (0.44–3.7)
>7				
Male	6 (2)	23 (2)	0.86 (0.32–2.3)	0.82 (0.28–2.4)
Female	1 (1)	11 (2)	0.22 (0.03–1.8)	0.15 (0.02–1.5)

Oral contraceptives¶▴				
No	172 (88)	638 (94)	1.0 Referent	1.0 Referent
Yes	23 (12)	41 (6)	2.4 (1.2–4.8)	2.0 (0.91–4.5)

Aspirin (# daily pills)*۸¶				
≤6				
Male	316 (98)	994 (98)	1.0 Referent	1.0 Referent
Female	191 (98)	664 (98)	1.0 Referent	1.0 Referent
>6				
Male	5 (2)	17 (2)	1.1 (0.35–3.2)	1.0 (0.31–3.3)
Female	4 (2)	15 (2)	0.76 (0.20–2.9)	0.69 (0.15–3.1)

Cortisone type medication¶				
No				
Male	310 (97)	965 (95)	1.0 Referent	1.0 Referent
Female	185 (95)	644 (95)	1.0 Referent	1.0 Referent
Yes				
Male	11 (3)	46 (5)	0.80 (0.40–1.6)	0.78 (0.36–1.7)
Female	10 (5)	35 (5)	1.2 (0.53–2.6)	1.1 (0.45–2.6

Ultraviolet radiation§				
No				
Male	312 (97)	1005 (99)	1.0 Referent	1.0 Referent
Female	193 (99)	675 (99)	1.0 Referent	1.0 Referent
Yes				
Male	9 (3)	6 (1)	4.4 (1.5–13)	5.0 (1.7–15)
Female	2 (1)	4 (1)	3.0 (0.51–18)	2.5 (0.34–19)

Dusts (asbestos, cement, or grain)§				
No				
Male	311 (97)	942 (93)	1.0 Referent	1.0 Referent
Female	195 (100)	677 (100)	1.0 Referent	1.0 Referent
Yes				
Male	10 (3)	69 (7)	0.41 (0.20–0.83)	0.40 (0.19–0.87)
Female	0 (0)	2 (0)	---+	---+

* Controls individually matched by gender, postal zip code of residence at time of diagnosis, birth year within 2 years, and year of MHCQ within 5 years.

٤ Adjusted for all other variables in table.

۶ Unknown category not shown.

† Defined as “Hobby, TV, *etc.*”

⋄ Defined as “Walking, sports, *etc.*”

~ Physician diagnosed.

* Including Aspirin, Empirin, Anacin, or Bufferin.

۸ Approximate usage on daily basis .

¶ Used in the past year.

§ Often or daily around at place of work.

▴ Women only.

+ Not computed due to zero referent cell.
